# Supplementary material for: RNA G-quadruplex structure contributes to cold adaptation in plants
Source: Nat Commun. 2022 Oct 20;13:6224. doi: 10.1038/s41467-022-34040-y (PMC9585020; doi:10.1038/s41467-022-34040-y)
Supplement: Supplementary file 1 — Supplementary Information [file 41467_2022_34040_MOESM1_ESM.pdf]

**Supplementary Information for**

# RNA G-quadruplex structure contributes to cold adaptation in plants

Xiaofei Yang[1,2,3,4]†, Haopeng Yu[1,4]†, Susan Duncan[4], Yueying Zhang[4], Jitender Cheema[4], Haifeng Liu[4,5], J. Benjamin Miller[6], Jie Zhang[4], Chun Kit Kwok[7,8], Huakun Zhang[1]*, Yiliang Ding[4]*

1. Key Laboratory of Molecular Epigenetics of Ministry of Education, Northeast Normal University, Changchun 130024, China
2. National Key Laboratory of Plant Molecular Genetics, CAS Center for Excellence in Molecular Plant Sciences, Institute of Plant Physiology and Ecology, Chinese Academy of Sciences, Shanghai 200032, China.
3. CAS-JIC Center of Excellence for Plant and Microbial Sciences, Institute of Plant Physiology and Ecology, Chinese Academy of Sciences, Shanghai 200032, China.
4. Department of Cell and Developmental Biology, John Innes Centre, Norwich Research Park, Norwich NR4 7UH, United Kingdom
5. State Key Laboratory of Crop Biology, College of Agronomy, Shandong Agricultural University, Taian 271018, China
6. School of Biological Sciences, University of East Anglia, Norwich Research Park, Norwich NR4 7TJ, United Kingdom
7. Department of Chemistry and State Key Laboratory of Marine Pollution, City University of Hong Kong, Kowloon Tong, Hong Kong SAR, China
8. Shenzhen Research Institute of City University of Hong Kong, Shenzhen 518057, China

* Correspondence to: Yiliang Ding (yiliang.ding@jic.ac.uk) and Huakun Zhang (zhanghk045@nenu.edu.cn)

† These authors contributed equally to this work.

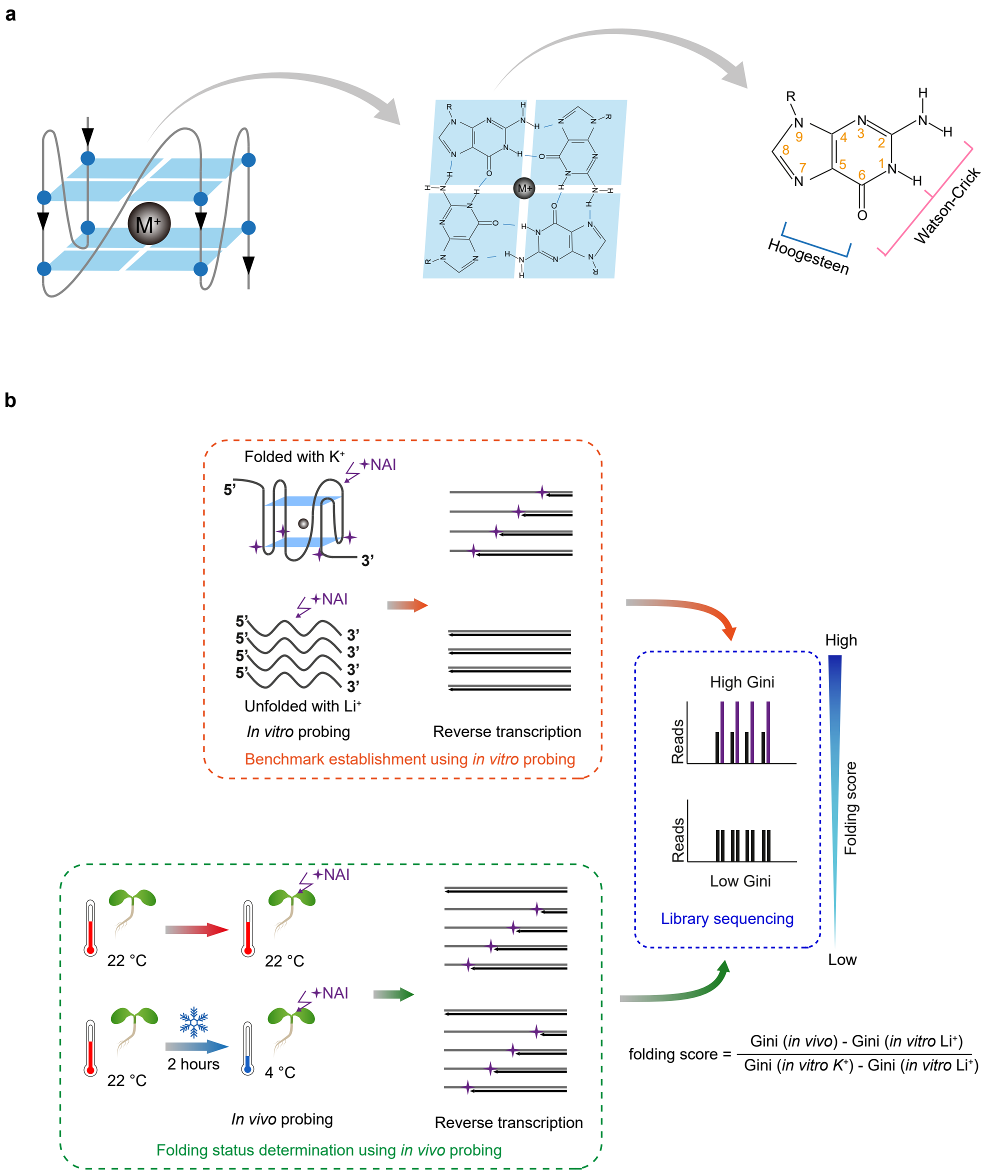


**Supplementary Fig. 1 | Schematic of RNA G-quadruplex structure and SHALiPE-seq method for RNA G-quadruplex detection *in vivo* at 22°C and 4°C.**

**(a)** Schematic illustration of RNA G-quadruplex structure. The blue and red lines on the guanine nucleotide indicate the Hoogesteen face or Watson-Crick face for RG4 formation respectively.

**(b)** Benchmarks of folded and unfolded RNA G-quadruplex (RG4) were initially established *in vitro* (top panel) in the presence of $K^+$, which stabilizes RG4 folding, or in the presence of $Li^+$, which destabilizes RG4 folding. Using the SHALiPE *in vivo* probing method (lower panel), the NAI chemical (indicated by a purple star) specially modifies the last G of the G-tract of folded RG4s, therefore causing high counts of reverse transcription (RT) stops with an uneven distribution, resulting in a high Gini index (middle panel on the right). While for unfolded RG4s, RT stops show an even distribution and low Gini index. For RG4 detection at

22°C and 4°C *in vivo*, NAI modification was carried out at the corresponding temperature, followed by reverse transcription, library generation and sequencing. The reads distribution and Gini index at 22°C and 4°C *in vivo* were compared to that of $K^+$ *in vitro*, or $Li^+$ *in vitro*, respectively.

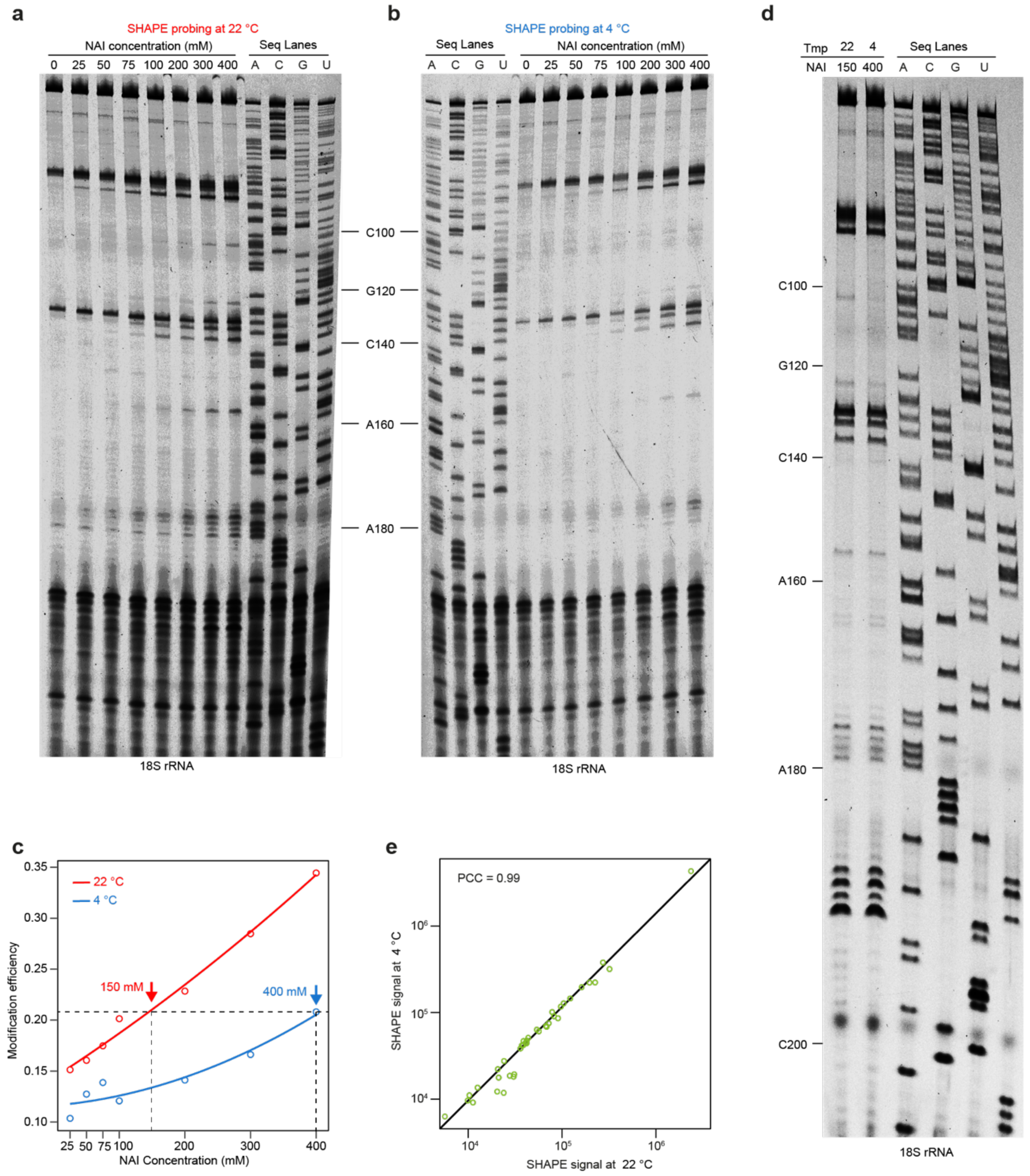


**Supplementary Fig. 2 | Comparable NAI modification in *Arabidopsis* at 22°C and 4°C.**

**(a** and **b)** Gel-based analysis of 18S rRNA for chemical probing of *Arabidopsis* at 22°C and 4°C, respectively.

**(c)** Modification curves of NAI on 18S rRNA at 22°C and 4°C. Modification efficiency was calculated by normalizing the signal of modified nucleotides to that of the whole lane in Supplementary Fig. 2a and Supplementary Fig. 2b.

**(d** and **e)** Comparison of chemical probing *in vivo* at 22°C and 4°C. Gel-based analysis shows comparable NAI probing profile of 18S rRNA (**d**) and high Pearson Correlation Coefficient (**e**, PCC = 0.99), with concentrations of 150mM NAI at 22°C and 400 mM NAI at 4°C, as determined in Supplementary Fig. 2c.

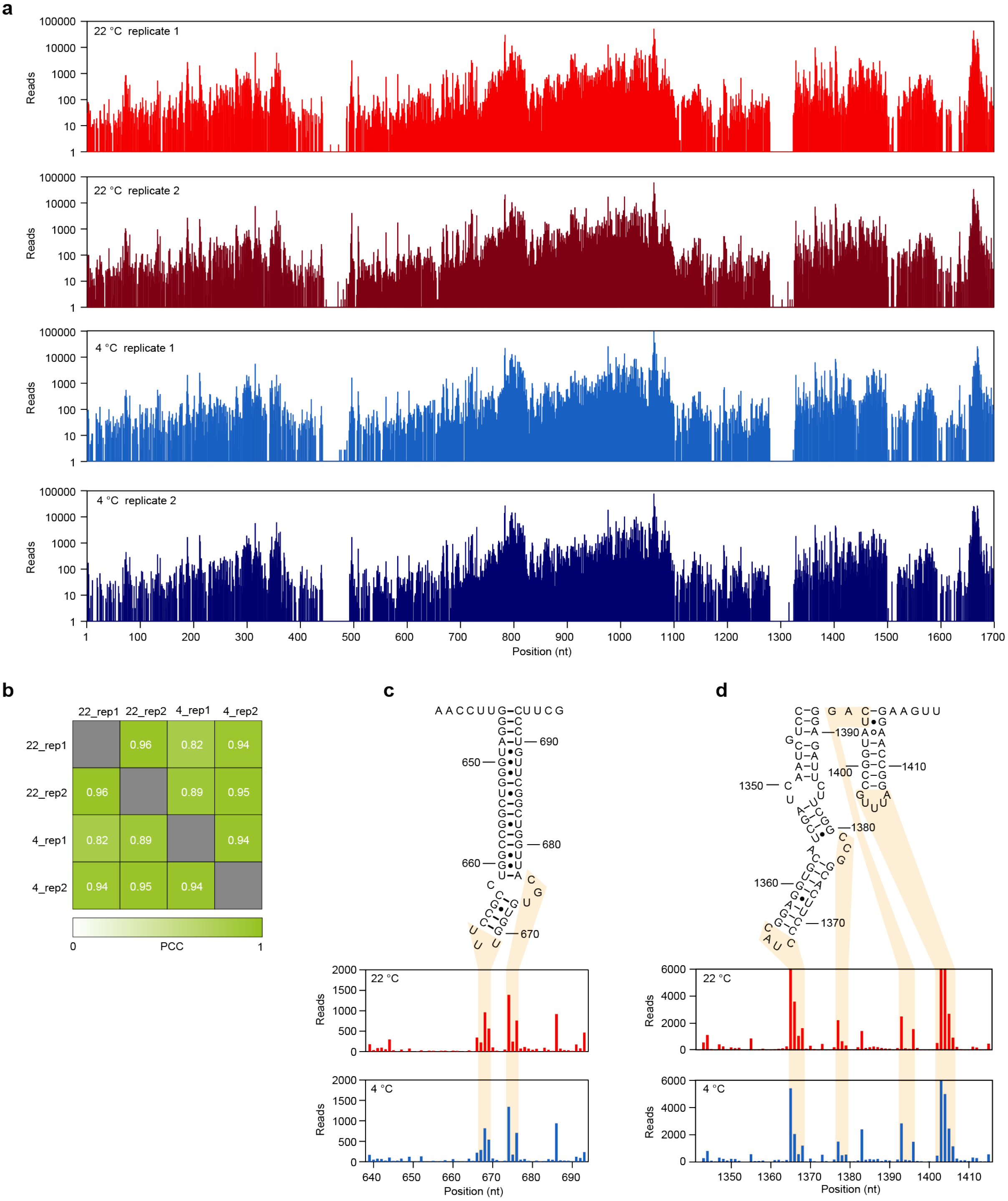

**Supplementary Fig. 3 | Robust and accurate determination of RNA structure using SHALiPE-seq at 22°C and 4°C.**

**(a)** The RT-stops of SHALiPE-seq on each nucleotide of 18S rRNA at 22°C and 4°C.

**(b)** High correlations of RT-stops between independent biological replicates and different temperatures.

**(c** and **d)** High agreement between SHALiPE-seq profile and phylogenetic structure on 18S rRNA. The unpaired regions in the phylogenetic structure showing high RT-stops at 22°C and 4°C are highlighted by light orange.

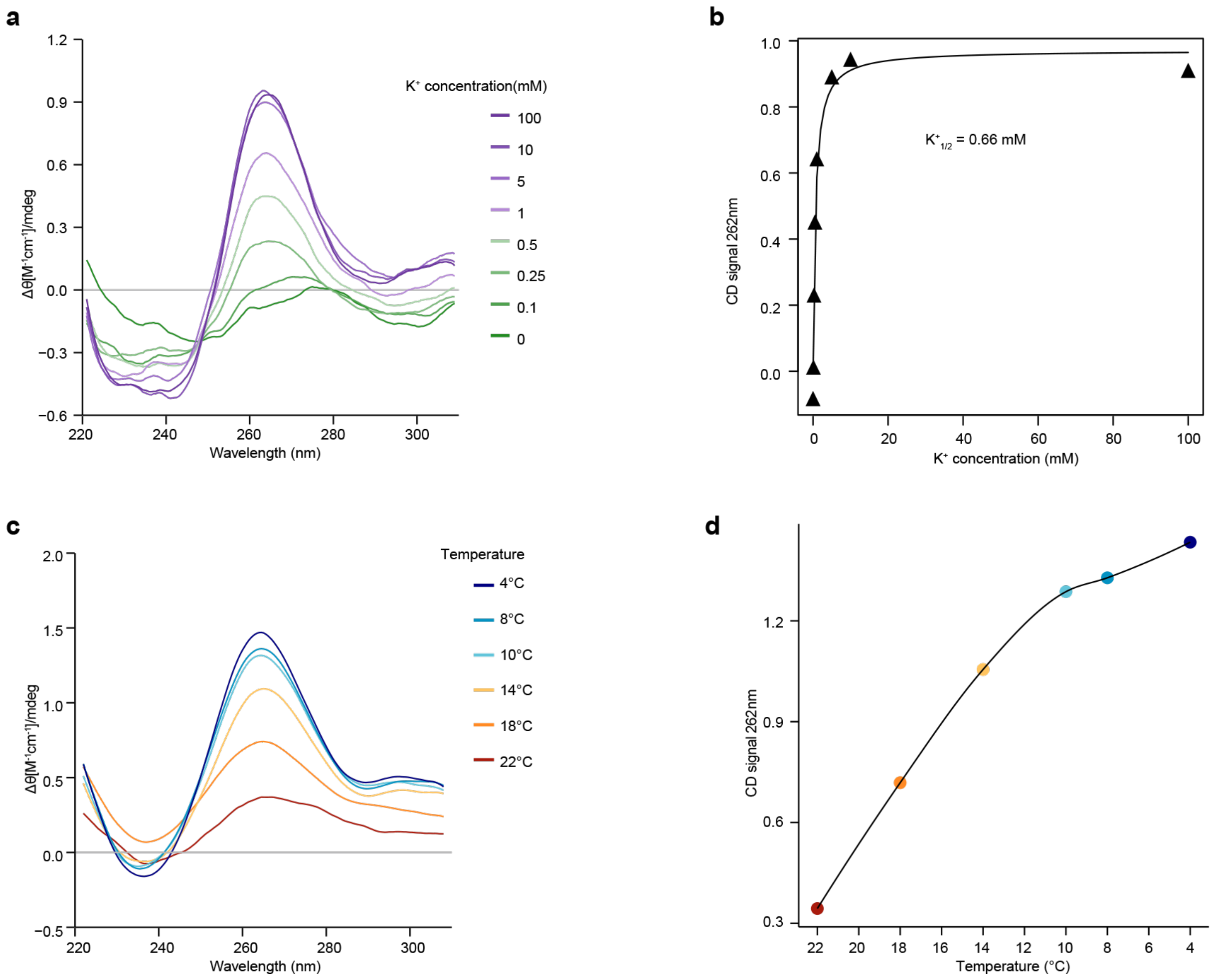


**Supplementary Fig. 4 | Biophysical characterization for the folding status of the RG4s on *AT3G20470*.**

**(a)** CD spectra as a function of $K^+$ concentration. $K^+$ ion-induced RG4 folding is at 2.5μM RNA in a background of 10 mM lithiumcacodylate (LiCac) (pH 7.0), as KCl concentration is increased up to 100mM. Both the positive peak at ~260 nm and the negative peak at ~240 nm were enhanced with the rise of $K^+$ concentration.

**(b)** CD signal (ellipticity monitored at 262 nm) as a function of $K^+$ concentration from 0 to 100mM (normal cellular condition) shows strong folding status of this RG4.

**(c)** CD spectra as a function of reducing temperature. The temperatures were reduced from 22°C to 4°C where both the positive peak at ~260 nm and the negative peak at ~240 nm were enhanced.

**(d)** CD signal (ellipticity monitored at 262 nm) as a function of temperature declines from 22°C to 4°C shows distinct folding status transitions in RG4 folding.

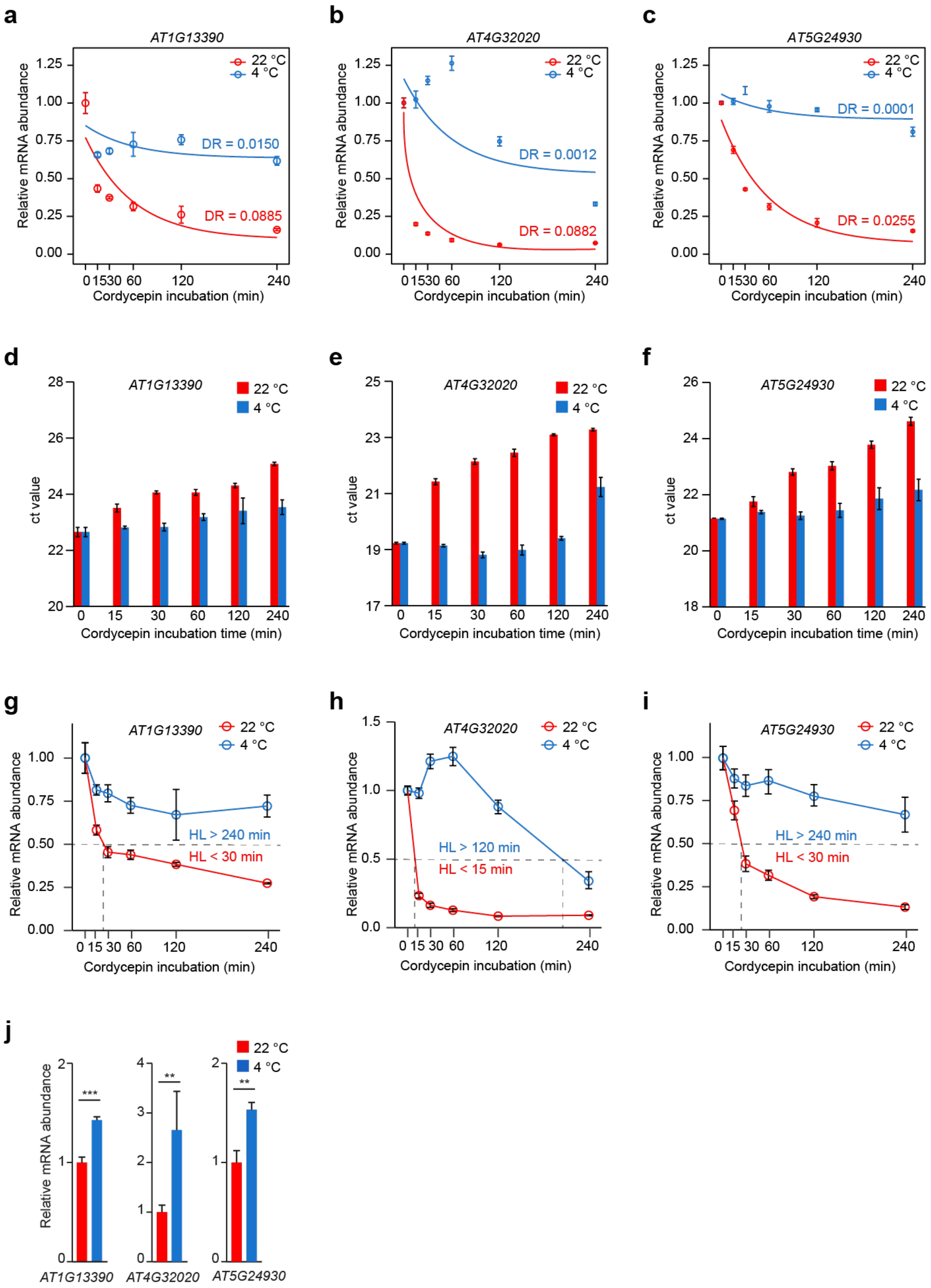


**Supplementary Fig. 5 | RG4 in 3'UTR represses mRNA decay and increases mRNA abundance in cold.**

**(a-c)** Line plot showing RNA abundance profiles with transcriptional arrest at 22°C or 4°C for selected transcripts *AT1G13390* **(a)**, *AT4G32020* **(b)** and *AT5G24930* **(c)** with cold-responsive RG4 in 3'UTRs. For these transcripts, the decay rate (DR) was significantly smaller at 4°C compared to that at 22°C, indicating slower mRNA decay in the cold. Data were extracted from RNA-seq experiments and are presented as mean values ± SE of 3 biological replicates.

**(d-f)** Validation of RNA decay using qRT-PCR for selected transcripts at 22°C and 4°C. For *AT1G13390* (**d**), *AT4G32020* **(e)** and *AT5G24930* **(f)**, ct values strongly increased according to transcriptional arrest duration at 22°C, suggesting a significant decrease of mRNA abundance with high decay rates at 22°C. The ct values at 4°C increased very slowly, suggesting high stability of mRNA abundance with slow decay at 4°C. Data are presented as mean values ± SE.

**(g-i)** Line plot showing RNA abundance profiles of selected transcripts with transcriptional arrest at 22°C or 4°C. Relative mRNA abundance of *AT1G13390* (**g**), *AT4G32020* (**h**) and *AT5G24930* (**i**) was normalized to the RNA abundance before transcriptional arrest, using the data shown in Supplementary Fig. 5d-f. *AT1G13320 (PP2A)* was used as an internal control. Data are presented as mean values ± SE of 3 biological replicates. HF: half-life.

**(j)** Comparison of mRNA abundance at 22°C and 4°C for selected transcripts with cold-responsive RG4 in 3'UTR. RNA abundance was normalized to internal control of *AT1G13320*. Data are presented as mean values ± SE of 3 biological replicates. ** $P < 0.01$ and ** $P < 0.001$ by one-sided Student's t-test.

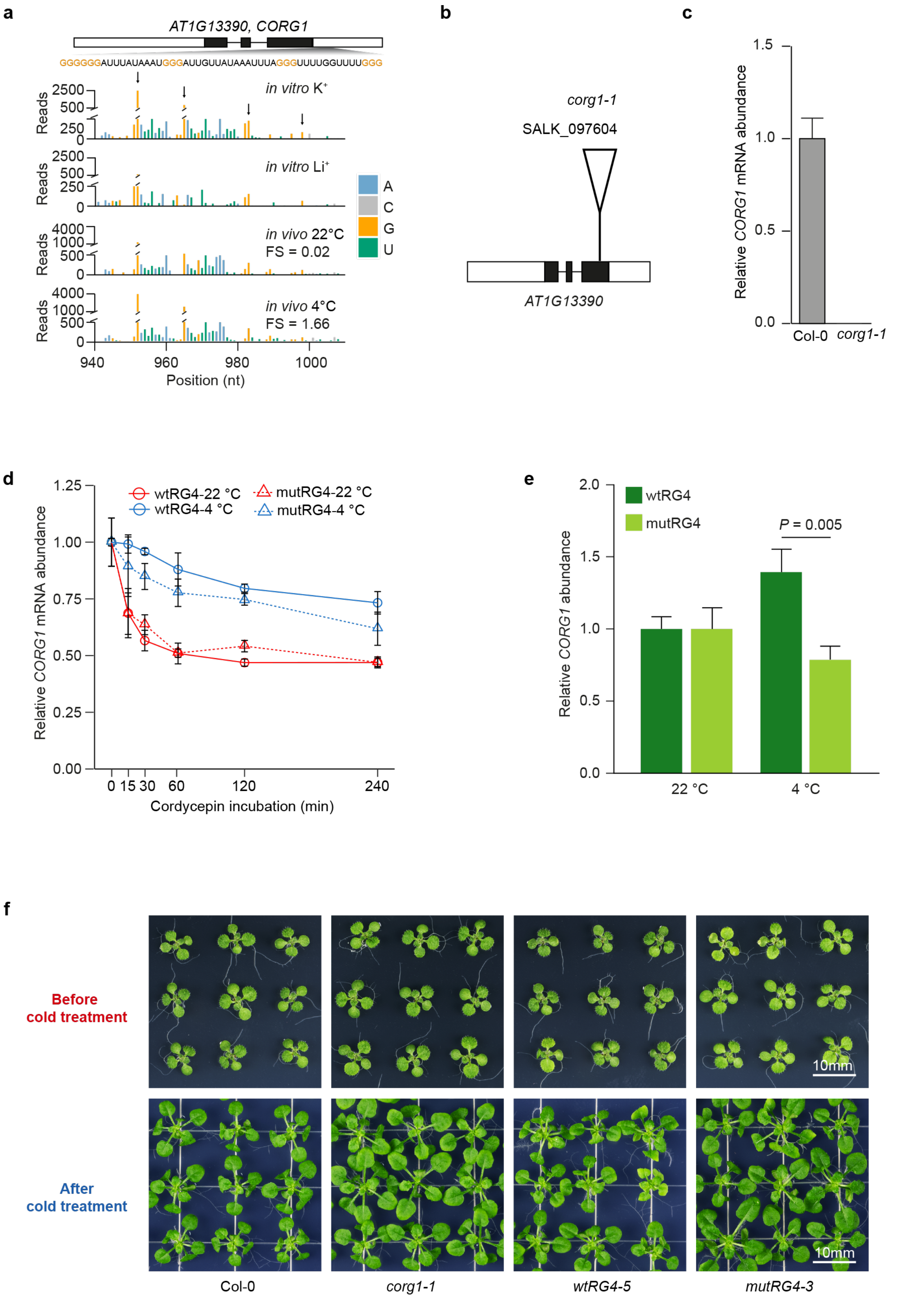


**Supplementary Fig. 6 | RNA G-quadruplex contributes to thermo-dependent mRNA stability and plant growth.**

**(a)** SHALiPE-seq profiles of the RG4s in 3'UTR of *AT1G13390* (*CORG1*). High reads on last Gs (dark arrow) are shown *in vitro* with $K^+$, compared to that *in vitro* with $Li^+$. *In vivo* profiles

show unfolded state at 22°C resembles that with $Li^+$, but folded state at 4°C resembles that with $K^+$. FS: folding score.

**(b)** Schematic diagram of *CORG1* showing the T-DNA insertion site of *corg1-1* (SALK_097604).

**(c)** Relative mRNA abundance of *CORG1* in Col-0 and *corg1-1* plants indicating *corg1-1* is a null mutant. Data are presented as mean values and SE of 3 biological replicates.

**(d)** RNA decay curves at 22°C and 4°C for endogenous *CORG1* transcripts with 3'UTR carrying wtRG4 or mutRG4. Relative mRNA abundance was normalized to the RNA abundance before transcriptional arrest with cordycepin incubation, according to internal control of *PP2A*. Data are presented as mean values ± SE of 4 biological replicates.

**(e)** Relative mRNA abundance of *CORG1* at 22°C and 4°C in complemented *corg1-1* mutant using genomic DNA sequence of *CORG1* carrying wtRG4 or mutRG4 in 3'UTR, respectively. Data are presented as mean values ± SE of 3 biological replicates.

**(f)** Phenotypes of plants of different genotypes grown at 22°C or 4°C. wtRG4 or mutRG4 denotes *corg1-1* mutant complemented with genomic DNA of *CORG1* carrying wtRG4 or mutRG4 respectively, as illustrated in Fig. 4a. Comparison was performed on the whole plant grown at 22°C for 1 week, or at 4°C for 4 weeks.

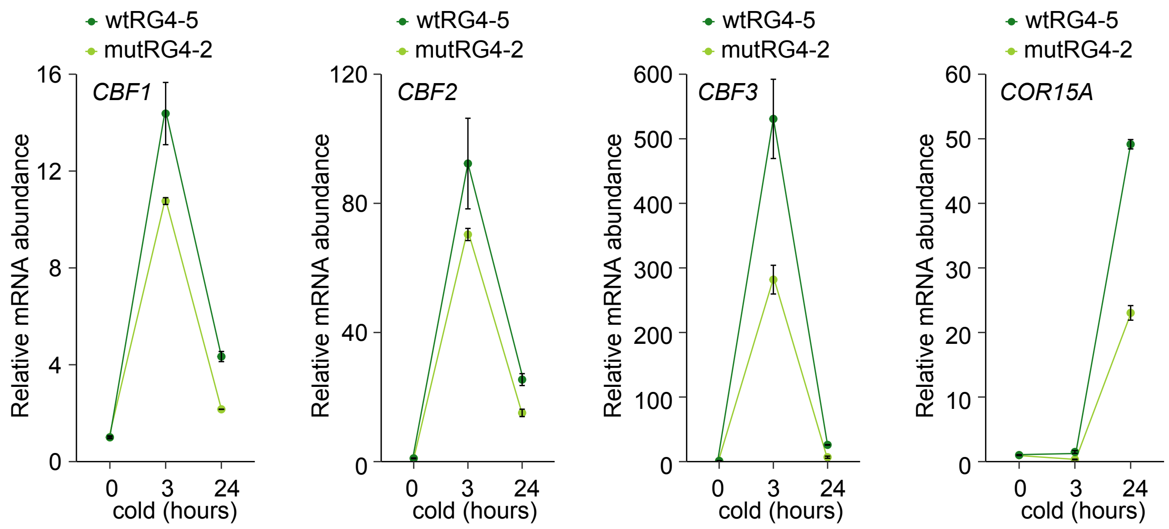


**Supplementary Fig. 7 | RNA G-quadruplex contributes to cold responsive gene expression.**

Relative mRNA abundances of marker genes *CBF1*, *CBF2*, *CBF3* and *COR15A* upon cold treatment (0, 3, 24 hours) in complemented *corg1-1* mutant using genomic DNA sequence of *CORG1* carrying wtRG4 or mutRG4 in 3'UTR, respectively. Error bar indicates SE of 4 biological replicates.
