# Supplementary material for: RNA G-quadruplex structure contributes to cold adaptation in plants
Source: Nat Commun. 2022 Oct 20;13:6224. doi: 10.1038/s41467-022-34040-y (PMC9585020; doi:10.1038/s41467-022-34040-y)
Supplement: Supplementary file 3 — Description of Additional Supplementary Files [file 41467_2022_34040_MOESM3_ESM.pdf]

Description of Additional Supplementary Files

File Name: Supplementary Data 1
Description: Nucleotide frequency of transcriptomes of land plants from the One Thousand Plant Transcriptomes Initiative (1KP)

File Name: Supplementary Data 2
Description: Geographic and bioclimatic features for land plants from the One Thousand Plant Transcriptomes Initiative (1KP)

File Name: Supplementary Data 3
Description: RG4 density in land plants from the One Thousand Plant Transcriptomes Initiative (1KP)

File Name: Supplementary Data 4
Description: Folding score of RG4s at 22°C and 4°C in Arabidopsis

File Name: Supplementary Data 5
Description: RNA-seq and Polysome-seq at 22°C and 4°C in Arabidopsis

File Name: Supplementary Data 6
Description: mRNA decay rate at 22°C and 4°C in Arabidopsis Table

File Name: Supplementary Data 7
Description: Plant genotypes and primers used in this study
