# Supplementary material for: RNA G-quadruplex structure contributes to cold adaptation in plants
Source: Nat Commun. 2022 Oct 20;13:6224. doi: 10.1038/s41467-022-34040-y (PMC9585020; doi:10.1038/s41467-022-34040-y)
Supplement: Supplementary file 11 — Reporting Summary [file 41467_2022_34040_MOESM11_ESM.pdf]

nature portfolio

Corresponding author(s): YILIANG DING

Last updated by author(s): Oct 03, 2022

Reporting Summary

Nature Portfolio wishes to improve the reproducibility of the work that we publish. This form provides structure for consistency and transparency in reporting. For further information on Nature Portfolio policies, see our Editorial Policies and the Editorial Policy Checklist.

Statistics

For all statistical analyses, confirm that the following items are present in the figure legend, table legend, main text, or Methods section.

n/a	Confirmed	
☐	☒	The exact sample size (*n*) for each experimental group/condition, given as a discrete number and unit of measurement
☐	☒	A statement on whether measurements were taken from distinct samples or whether the same sample was measured repeatedly
☐	☒	The statistical test(s) used AND whether they are one- or two-sided<br>*Only common tests should be described solely by name; describe more complex techniques in the Methods section.*
☒	☐	A description of all covariates tested
☒	☐	A description of any assumptions or corrections, such as tests of normality and adjustment for multiple comparisons
☐	☒	A full description of the statistical parameters including central tendency (e.g. means) or other basic estimates (e.g. regression coefficient) AND variation (e.g. standard deviation) or associated estimates of uncertainty (e.g. confidence intervals)
☐	☒	For null hypothesis testing, the test statistic (e.g. *F*, *t*, *r*) with confidence intervals, effect sizes, degrees of freedom and *P* value noted<br>*Give P values as exact values whenever suitable.*
☒	☐	For Bayesian analysis, information on the choice of priors and Markov chain Monte Carlo settings
☐	☒	For hierarchical and complex designs, identification of the appropriate level for tests and full reporting of outcomes
☐	☒	Estimates of effect sizes (e.g. Cohen's *d*, Pearson's *r*), indicating how they were calculated

Our web collection on statistics for biologists contains articles on many of the points above.

Software and code

Policy information about availability of computer code

Data collection	The raw sequencing data collection was performed by BGI Genomics Co.Ltd
Data analysis	For RNA-seq and polysome-seq libraries, reads were directly used for alignment. For SHALiPE-seq libraries, the first 3 bases at the 5′ end (random nucleotides on adaptor for cDNA ligation) of the raw reads were cropped. The alignment was carried out against Arabidopsis transcriptome TAIR10 release using bowtie version 1.0.1 with iterative mapping procedure. The minimum read length allowed to map was fixed to 21 bases long. The resulting mapped sam files were converted to bam files and indexed using samtools-1.4.1. The stop counts were extracted using HTseq v0.7.2 and the code was written in Python v2.7.15. Reads counts of replicates were merged after observing high correlations amongst these.

For manuscripts utilizing custom algorithms or software that are central to the research but not yet described in published literature, software must be made available to editors and reviewers. We strongly encourage code deposition in a community repository (e.g. GitHub). See the Nature Portfolio guidelines for submitting code & software for further information.

Data

Policy information about availability of data

All manuscripts must include a data availability statement. This statement should provide the following information, where applicable:

- Accession codes, unique identifiers, or web links for publicly available datasets
- A description of any restrictions on data availability
- For clinical datasets or third party data, please ensure that the statement adheres to our policy

The raw data have been deposited in the Sequence Read Archive (SRA) (https://www.ncbi.nlm.nih.gov/sra) under the BioProject ID number PRJNA762705. SHALiPE-seq data in vitro and in vivo at 22 ℃ are available under BioProject ID number PRJNA561194 (Yang et al., 2020). The processed data are provided in the Supplementary Tables S1-S7. Resources for the latitude and longitude of plant geographic distribution obtained from observations collected by the Global Biodiversity Information Facility (www.gbif.org) are listed in Table S2, i.e. Arabidopsis thaliana: 10.15468/dl.pmy46j. WorldClim database could be accessed through the server https://worldclim.org/.

Human research participants

Policy information about studies involving human research participants and Sex and Gender in Research.

Reporting on sex and gender	NA
Population characteristics	NA
Recruitment	NA
Ethics oversight	NA

Note that full information on the approval of the study protocol must also be provided in the manuscript.

Field-specific reporting

Please select the one below that is the best fit for your research. If you are not sure, read the appropriate sections before making your selection.

☒ Life sciences ☐ Behavioural & social sciences ☐ Ecological, evolutionary & environmental sciences

For a reference copy of the document with all sections, see nature.com/documents/nr-reporting-summary-flat.pdf

Life sciences study design

All studies must disclose on these points even when the disclosure is negative.

Sample size	Sample sizes were determined based on previous publications on similar experiments. The determined sample size was adequate as the differences between experimental groups was significant and reproducible
Data exclusions	No data were excluded from analysis
Replication	The RNA structure experiments were performed with two independent biological replicates. The polysome-seq and transcription-arrest experiments were performed with 3 biological replicates. All key experimental findings in the structural mutation analysis were reproduced in more than three independent biological replicates.
Randomization	For RNA structure experiments, hundreds of seedlings from multiple plates were collected for each replicate. For RNA expression analysis, randomly selected plants from a plate were collected. For reporter assay, tobacco plants were randomly chosen for Agrobacterium infiltration with different vectors. For bioimaging, seedlings were randomly chosen for further experiments with different treatment.
Blinding	Blinding was not necessary for the molecular biology techniques, where bias could not be introduced as samples were treated together and identically. For bioimaging, the same settings were used for all comparisons.

Reporting for specific materials, systems and methods

We require information from authors about some types of materials, experimental systems and methods used in many studies. Here, indicate whether each material, system or method listed is relevant to your study. If you are not sure if a list item applies to your research, read the appropriate section before selecting a response.

Materials & experimental systems

n/a	Involved in the study
☐	☒ Antibodies
☒	☐ Eukaryotic cell lines
☒	☐ Palaeontology and archaeology
☒	☐ Animals and other organisms
☒	☐ Clinical data
☒	☐ Dual use research of concern

Methods

n/a	Involved in the study
☒	☐ ChIP-seq
☒	☐ Flow cytometry
☒	☐ MRI-based neuroimaging

Antibodies

Antibodies used	BG4 antibody, His-Tagged, Lambda antibody AB00174-1.6, Absolute Antibody
Validation	Specificity: Binds with high selectivity and low nanomolar affinity to DNA and RNA G-quadruplex structures. Published Application(s): EMSA, ELISA, IF; Published Species Reactivity: Human
